# Preparing real-world data through common data model harmonization of cancer patient records in the COMNet platform at the Modena Oncology Center

**DOI:** 10.3389/fdgth.2026.1760649

**Published:** 2026-04-09

**Authors:** Luca Moscetti, Enrico Calanchi, Elisa Pettorelli, Andrea Spallanzani, Federica Bertolini, Rossella Fogliani, Mirko Orsini, Laura Delsante, Monica Civallero, Roberta Depenni, Katia Di Emidio, Fabio Gelsomino, Annalisa Fontana, Federico Piacentini, Roberto Sabbatini, Massimo Dominici

**Affiliations:** 1 Division of Medical Oncology, Department of Oncology and Hematology, University Hospital of Modena, Modena, Italy; 2 Datariver, Modena, Italy; 3 Servizio Tecnologie dell’informazione, University Hospital of Modena, Modena, Italy; 4 Division of Medical Oncology, Department of Medical and Surgical Sciences for Children and Adults, University Hospital of Modena, Modena, Italy; 5 Department of Oncology and Hematology, Azienda Ospedaliero-Universitaria di Modena, Modena, Italy

**Keywords:** data harmonization, electronic health records, OMOP Common Data Model, oncology, real-world data

## Abstract

**Objectives:**

The transition from paper medical records to electronic health records (EHRs) has enabled the extraction of substantial real-world data, which can support future real-world evidence generation. This study aimed to convert heterogeneous oncology data from local EHR systems—collectively referred to as COMNet—into a standardized data model. In particular, the Observational Medical Outcomes Partnership Common Data Model (OMOP-CDM) was adopted to harmonize routinely collected clinical data into a common database, thereby enabling standardized secondary use and large-scale analyses.

**Methods:**

Demographic and clinical parameters routinely collected at the Modena Cancer Center were retrospectively extracted from COMNet and harmonized into the OMOP-CDM through an Extract–Transform–Load process supported by the European Health Data and Evidence Network (EHDEN).

**Results:**

We identified 85,026 persons with at least one recorded condition occurrence. Discrepancies were observed across OMOP-CDM domains—particularly in visit occurrence and drug exposure—reflecting changes in documentation practices and source systems over time. The temporal trend of data migration to the electronic platform revealed two significant peaks, corresponding to initial data entry and subsequent digitalization of hospital facilities and pharmacy records.

**Discussion:**

The harmonization process revealed data inconsistencies, including incompleteness and missing data, reflecting challenges inherent in the transition from paper-based records to electronic systems and the coexistence of different legacy software platform.

**Conclusions:**

Harmonizing COMNet data into the OMOP-CDM produced a standardized real-world data resource that can support future observational research and participation in federated network studies. Ongoing initiatives, including the EHDEN project, are supporting the further development of COMNet to improve interoperability and enhance structured data capture.

## Background

The evolution of treatments—particularly the development of targeted therapies and the advent of immunotherapy—has revolutionized therapeutic strategies for early and metastatic cancers over the past decade (1, 2). The availability of new treatments has highlighted that individual patient clinical information alone is often insufficient to define optimal treatment, necessitating the integration of anatomical, pathological, and molecular parameters obtained through advanced technologies such as gene sequencing. The effectiveness of new drugs—both as monotherapy and in combination with chemotherapy or hormone therapy—is associated with various predictive factors derived from immunohistochemical and molecular biology analyses (3). The dynamic nature of this treatment landscape, shaped by registration trials with specific patient selection criteria, underscores the need to collect real-world data (RWD) to evaluate the feasibility and outcomes of these therapies in routine outpatient clinical practice (4). The transition from paper records to electronic health records (EHRs) has facilitated access to large volumes of clinical information potentially useful for observational research. However, the secondary use of EHR data is frequently limited by data quality issues such as incompleteness, inconsistencies, and heterogeneous coding practices (5–7).

To address these challenges, we conducted a retrospective study involving the extraction of available oncology data from the Modena Cancer Center, supported by the European Health Data and Evidence Network (EHDEN), a European initiative promoting the adoption of the OMOP Common Data Model (OMOP-CDM) across sites (8). The OMOP-CDM enables federated observational research across distributed data partners (9, 10). The OMOP-CDM was utilized to convert heterogeneous data from EHR sources within the local oncology data platform (COMNet) into a standardized structure and vocabulary, creating a common database that enables the secondary use of routinely collected clinical data (11, 12). This work contributes to the existing OMOP-CDM literature by describing a real-world implementation in an academic oncology center and highlighting practical challenges related to historical EHR migrations and data quality.

## Methods

This retrospective, single-center, observational cohort study included all patients with solid tumors who presented at the Modena Cancer Center from 2001 onward. The objective of the study was to create a database by collecting demographic and clinical parameters to evaluate the outcomes of patients treated in routine clinical practice, distinct from those in clinical trials.

This database was designed to support the development of future real-world evidence studies by enabling the identification of relevant clinical parameters and providing a standardized, anonymized data platform suitable for large-scale analyses.

For included patients, retrospective data collection encompassed the following:

- Demographic information: gender, age, place of birth (referring to the municipality and country of birth as recorded in the institutional administrative systems), and ethnicity.
- Clinical data: start and end dates of therapies, dates and results of instrumental re-evaluations (i.e., imaging-based reassessments performed during follow-up, such as CT, MRI, or PET, when available in structured form).
- Histopathological and molecular diagnoses.
- Therapies: systemic and locoregional treatments.

All prevalent and incident patients diagnosed with solid tumors from 2001 onward were consecutively enrolled.

Eligibility criteria were as follows:

- Age >18 years.
- Diagnosis of solid neoplasia since 2001 (eligibility determined by the presence of an oncology diagnosis recorded in the source systems and mapped into the OMOP condition occurrence domain).
- All disease stages of solid tumors managed by oncology for surgery, subsequent therapies, or follow-up.

Data were obtained from multiple sources, including institutional administrative databases, radiological examination databases, and laboratory databases. These systems were integrated through a unified patient identification process, with each individual assigned a unique ID code. Patient records from multiple institutional systems were linked using the institutional master patient index prior to anonymization and OMOP conversion.

The IT and Telematics Technologies Service performed patient extraction queries from the various applications and ensured anonymization. DataRiver, an EHDEN-certified SME, provided support for the mapping process, Extract–-Transform–-Load (ETL) development, and the setup of the working environment and tools to standardize data according to the OMOP Common Data Model.

Technically, harmonization with the OMOP-CDM involved custom data extraction from the Microsoft SQL Server (MSSQL) data source, which was imported into a dedicated PostgreSQL instance. The resulting integrated and reorganized dataset enabled a fully automated ETL process without the need for additional manual intervention.

Analysis and control tools provided by the EHDEN community allowed continuous verification of the effectiveness and consistency of the standardization process, supporting distributed studies with global data partners.

Source data profiling and ETL design were performed using the OHDSI open-source tools White Rabbit and Rabbit-in-a-Hat. White Rabbit was used to scan the COMNet source database to generate a profiling report describing tables, fields, and value distributions. Rabbit-in-a-Hat was then used to design and document the mapping logic required to transform the COMNet source fields into the OMOP-CDM target tables.

## Ethical considerations

The study was conducted in accordance with Good Clinical Practice guidelines established by the International Council for Harmonization and the provisions of the Declaration of Helsinki. Approval was granted by the local ethical committee (reference number Comitato Etico dell’Area Vasta Emilia Nord, nr 180/2022, 23 November 2023). Although the study was observational, informed consent was obtained from patients who were alive and able to understand and sign a written statement of consent. This included consent to participate in drug-free clinical trials, the use of biological material for scientific purposes, and the processing of personal data, in compliance with the Privacy Law (Italian Legislative Decree No. 196/2003).

For patients who were deceased or could not be located, consent was not required, in accordance with the General Authorization to Process Personal Data for Scientific Research Purposes (1 March 2012) issued by the Guarantor for the Protection of Personal Data (published in the Official Italian Gazette No. 72, 26 March 2012). Moreover, earlier ethical approval was obtained from the same ethics committee (Comitato Etico Area Vasta Emilia Nord, reference number 001282/20, dated 15 January 2020), covering activities related to data extraction and management.

## Results

A total of 89,211 persons were included in the OMOP database. Among them, 85,026 had at least one record in the condition occurrence domain and 39,230 had a defined observation period. The distribution of records across OMOP domains is summarized in Table 1.

**Table 1:** Number of records in all clinical data tables.

Table name	Count	N_Persons
Drug_exposure	1,288,618	13,998
Measurement	1,175,181	83,838
Observation	533,197	85,367
Condition_occurrence	299,048	85,026
Procedure_occurrence	285,436	80,167
Condition_era	275,151	85,026
Drug_era	137,140	13,994
Person	89,211	89,211
Observation_period	57,775	39,230
Visit_occurrence	46,586	15,923
Death	39,677	39,677
Specimen	0	0
Dose_era	0	0
Device_exposure	0	0
Visit_detail	0	0
Location	0	NA
Cost	0	NA
Care_site	0	NA
Note	0	0
Payer_plan_period	0	0
Provider	0	NA

Visit occurrence was reported in 15,923 patients a (event where persons engage with the healthcare system for a duration of time). Death was recorded for 39,677 patients. A procedure occurrence (records of activities or processes ordered by, or carried out by, a healthcare provider on the patient with a diagnostic or therapeutic purpose) was conducted in 80,167, whereas a measurement (structured values, numerical or categorical, obtained through systematic and standardized examination or testing of a person or person’s sample: laboratory tests, vital signs, and quantitative findings from pathology report) was recorded in 80,167. Drug exposure—ingested or otherwise introduced into the body—was reported in 13,998.

Discrepancies across OMOP domains (visit occurrence, drug exposure, procedure occurrence, and measurement) were observed, reflecting differences in documentation practices and source systems over time. In particular, differences in drug exposure may reflect limitations in the structured capture of oral therapies. The complete results are shown in Table 1.

**Figure 1:**
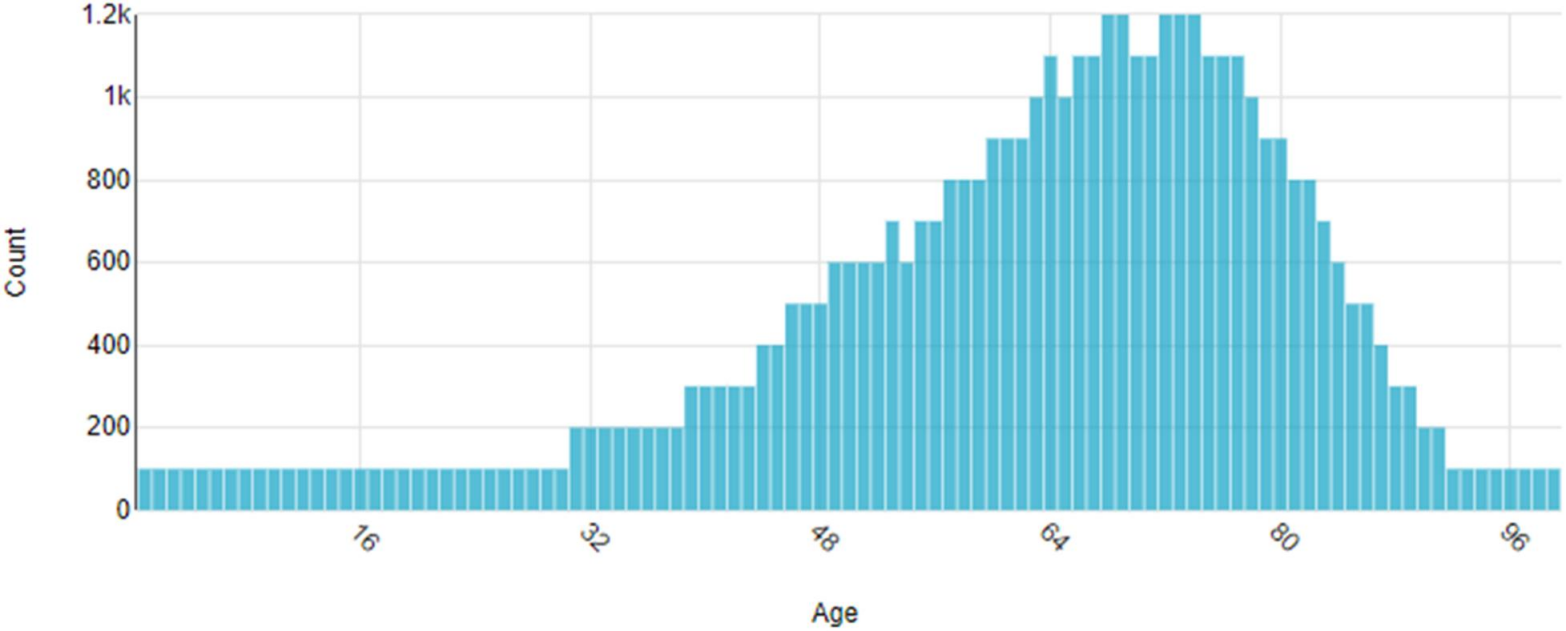
Distribution of patient age at first observation recorded in COMNet.

**Figure 2:**
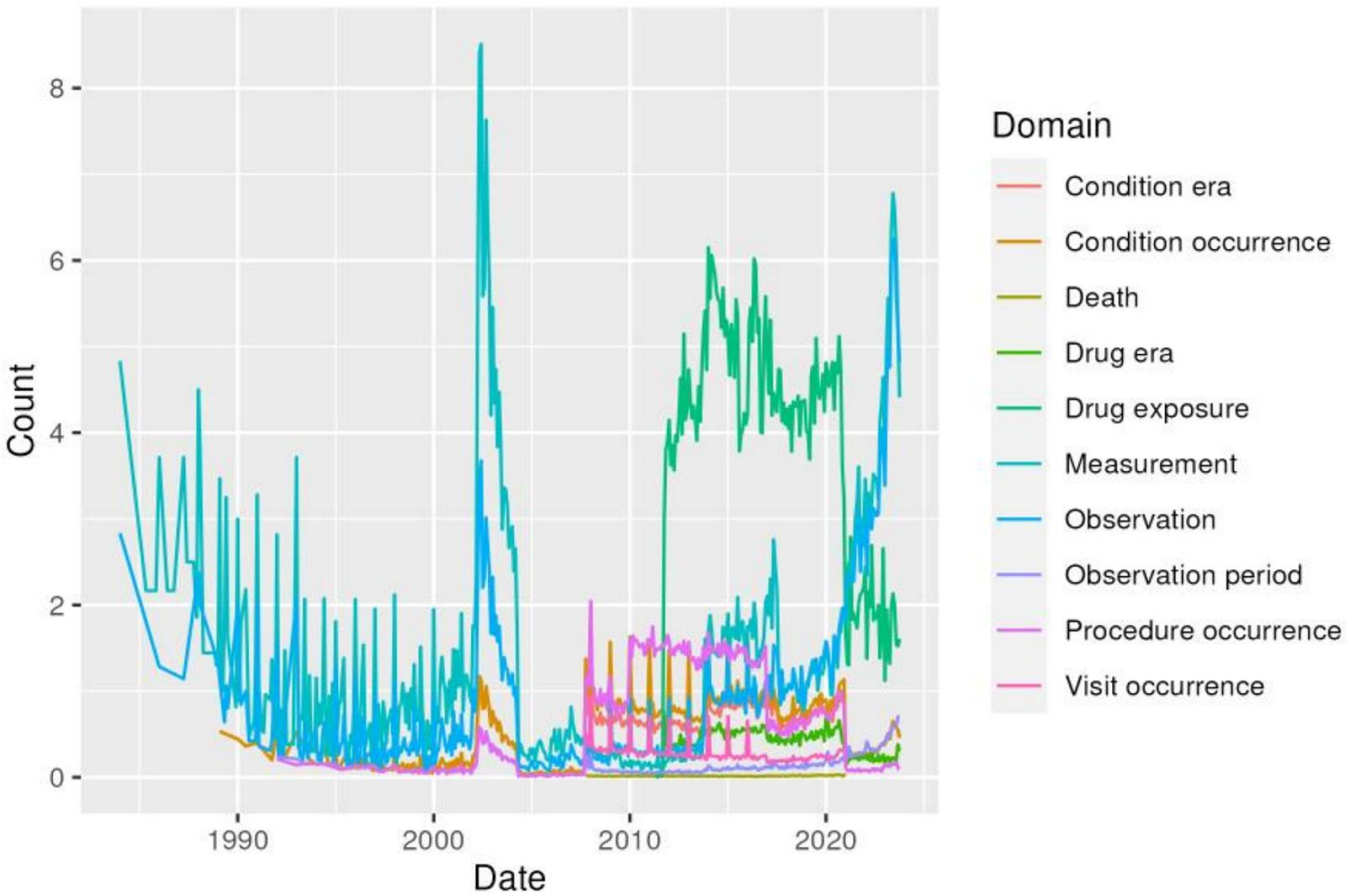
Data density plot showing the number of records per person over time across selected OMOP-CDM domains (condition occurrence, condition era, drug exposure, drug era, procedure occurrence, measurement, observation, visit occurrence, observation period, and death). Peaks in the curves reflect periods of increased data entry and progressive digitization of clinical information systems, including the integration of laboratory and pharmacy records.

Age at first observation is reported in Figure 1. The temporal trend of data migration to the electronic platform shows two significant peaks (Figure 2). The first peak occurred in the early 2000s with the entry of data related to observations and digitalization of laboratory assessment. A second peak was observed in the second decade of the 2000s, coinciding with the digitization of the hospital pharmacy (Figure 2).

**Figure 3:**
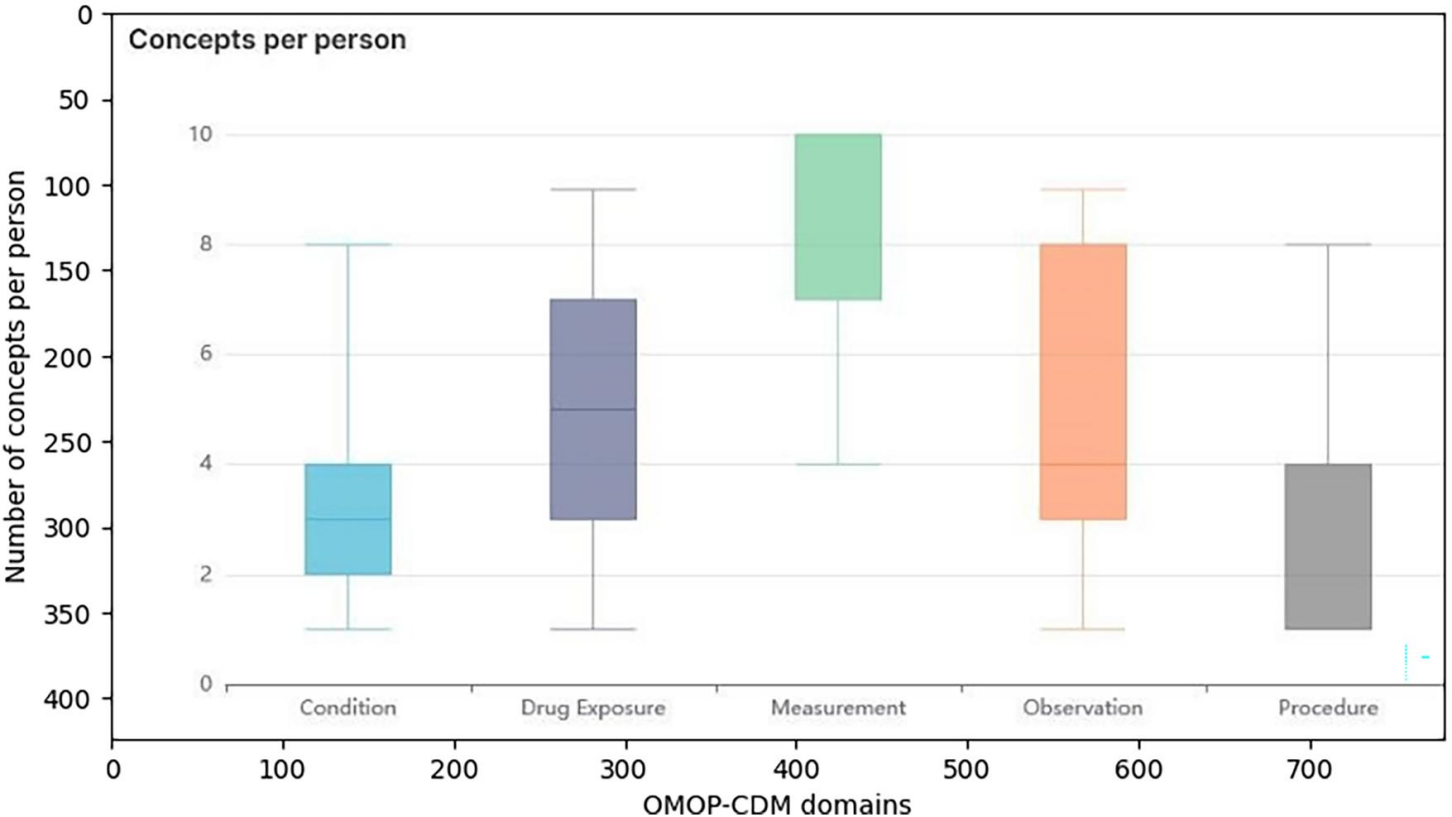
Boxplot showing the distribution of the number of distinct concepts per person across selected OMOP-CDM domains (Condition, Drug Exposure, Measurement, Observation, and Procedure). The plot illustrates median values and variability across domains.

The number of distinct concepts per person across OMOP domains is shown in Figure 3. The results highlight the increase in clinical data entry and digitalization over the last two decades. An increase in the use of cancer drugs was observed after 2010, reflecting the introduction of new systemic therapies, including targeted treatments and precision oncology approaches. In addition, a parallel increase in clinical instrumental controls was observed, reflected in the increased number of measurement and observation records. The limited availability of visit data prior to 2010 reflects changes in clinical documentation practices and the progressive digitalization of historical paper records collected during the long-standing activity of the Modena Oncology Center.

## Discussion

At our center, clinical data were collected on paper until 2002. Given the increasing complexity of oncology care and the growing volume of clinical information required for routine documentation, a gradual transition from paper medical records to an electronic chart (e-chart) was implemented. Supported by the EHDEN project, clinical information has been progressively migrated to the electronic platform over the last 20 years. However, data completeness and consistency were partially compromised by the heterogeneity of legacy systems and by the challenges inherent in repeated migrations.

The evolution of the clinical context, the type of information collected, and its organization required several updates of the initial mapping design and ETL scripts. In particular, additional staging and normalization layers were introduced between the source systems and the OMOP target tables to resolve inconsistencies across historical datasets and improve traceability. While these intermediate layers improved data coherence, they increased the complexity of the ETL pipeline and required additional development and validation time. The expansion of the data sources also led to substantial revisions of the SQL scripts used in the ETL process and extensions of the concept mapping.

In the updated dataset, multiple inconsistencies were identified, primarily related to missing or implausible dates (e.g., missing diagnosis dates, missing dates of information entry, or inconsistencies in therapy start/end timelines). Additional inconsistencies were observed in drug administration documentation, particularly regarding the number of therapeutic cycles and the longitudinal reconstruction of treatment exposure. Data cleansing activities and clarifications obtained from the source systems allowed improvements in the reconstruction of therapy duration and patient clinical history, achieving the best possible result from the available dataset.

Given the increasing relevance of biological and molecular characteristics in precision oncology, we aimed to collect and harmonize the clinical data of all patients treated at our center and transform them into a standardized structured data model through OMOP-CDM. This approach supports the secondary use of these data for research and quality improvement in clinical practice.

In terms of lessons learned, our experience highlights the importance of iterative source data profiling, traceability across historical migrations, and systematic plausibility checks on temporal variables and longitudinal treatment timelines.

Converting data derived from EHRs into OMOP-CDM can enhance health data governance by enabling standardized reuse of routinely collected clinical data for both healthcare and research purposes (13, 14). The adoption of EHRs has improved access to integrated clinical information and enabled the extraction of large volumes of data to evaluate clinical activity and support quality improvement initiatives. However, secondary use of EHR data for research is frequently limited by data quality issues and heterogeneous documentation practices (15, 16). Data quality harmonization represents a key step in improving the reliability of EHR-derived datasets (6).

As suggested by Weiskopf and Weng, EHR-derived data intended for secondary use require systematic quality assessment methodologies tailored to the research task (7). Core quality dimensions include completeness, correctness, plausibility, concordance, and currency. In our experience, the analysis of the COMNet dataset confirmed incompleteness, inconsistent and implausible data, and missing information. The temporal trends observed in the harmonized dataset also reflect progressive digitization over the last two decades, with increasing documentation of systemic therapies and instrumental controls after 2010, and with limited visit information in earlier years, consistent with changes in documentation workflows and EHR adoption over time.

Further use of routinely collected clinical data will be essential to expand research activities in academic centers. However, unmet needs remain, particularly the lack of dedicated research infrastructure to support sustainable data extraction, quality control, and reuse (16). The availability of high-quality RWD represents a fundamental prerequisite for generating real-world evidence (RWE) on the effectiveness and safety of medical products, including long-term outcomes and adverse events, and for evaluating complex procedures in settings where randomized clinical trials are not available (1, 4).

## Conclusion

This study describes the process of harmonizing routinely collected oncology data from our local EHR sources (COMNet) into the OMOP Common Data Model, to obtain a standardized dataset that can be used as RWD for future observational research. The resulting database represents a structured resource that can support analyses of routine clinical practice and may facilitate participation in collaborative network studies based on a common data model (17–20).

The work was supported by the EHDEN, a European initiative that promotes OMOP-CDM adoption across institutions to enable federated observational research (8). Our experience confirms that the progressive transition from paper records to electronic documentation—together with repeated historical migrations between different systems—can significantly affect data completeness and consistency. Iterative refinement of the ETL process and systematic data quality assessment are therefore essential steps to obtain the best possible result from the available clinical datasets and maximize the research value of EHR-derived oncology data.

## Data Availability

The original contributions presented in the study are included in the article/Supplementary Material, further inquiries can be directed to the corresponding author.
